# Imaging specific newly synthesized proteins within cells by fluorescence resonance energy transfer†
†Electronic supplementary information (ESI) available. See DOI: 10.1039/c6sc02610a


**DOI:** 10.1039/c6sc02610a

**Published:** 2016-09-12

**Authors:** Linfeng Sheng, Lesi Cai, Jie Liu, Sichun Zhang, Jing-Juan Xu, Xinrong Zhang, Hong-Yuan Chen

**Affiliations:** a State Key Laboratory of Analytical Chemistry for Life Science , Collaborative Innovation Center of Chemistry for Life Sciences , School of Chemistry and Chemical Engineering , Nanjing University , 210023 , China . Email: xujj@nju.edu.cn; b Department of Chemistry , Beijing Key Laboratory of Microanalytical Methods and Instrumentation , Tsinghua University , Beijing 100084 , China . Email: xrzhang@mail.tsinghua.edu.cn

## Abstract

Metabolic azide amino acid labelling followed by the use of bioorthogonal chemistry is an efficient technique for imaging newly synthesized proteins. Recently, AHA-labelling together with the proximity-ligation assay was used to identify newly synthesized proteins of interest (POI) (Tom Dieck *et al.*, *Nat. Meth.* 2015, **12**, 411). Here we build on this study replacing the proximity-ligation assay with FRET to improve the spatial resolution. Herein, we develop a FRET-based strategy for imaging the newly synthesized endogenous POI within cells: a FRET acceptor is installed onto the newly synthesized proteins *via* click chemistry, and a FRET donor onto the POI *via* immunocytochemistry. We found that a photobleaching based FRET efficiency imaging mode and a fluorescence lifetime imaging mode showed the distribution of newly synthesized proteins more accurately compared to the direct observation of FRET signals. We demonstrated the capability of this FRET-based imaging method by visualizing several newly synthesized proteins including TDP-43, tubulin and CaMKIIα in different cell lines. This novel analytical imaging method could be used to visualize other specific endogenous proteins of interest *in situ*.

## Abstract 2

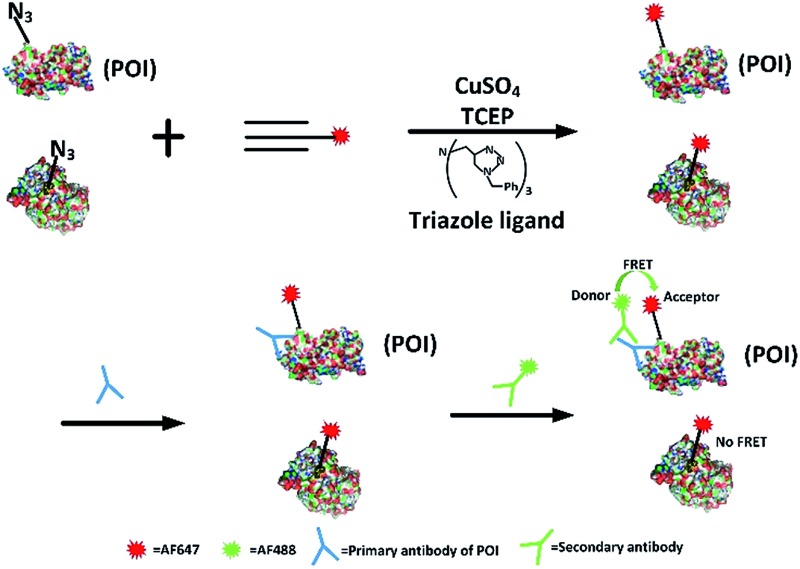
A FRET-based strategy is developed to image and track specific newly synthesized endogenous proteins *in situ*.

## Introduction

Both cell homeostasis and cellular response to environmental stimulus require spatiotemporally regulating protein synthesis. The synthesis and translational control of new proteins are necessary for many intricate biological processes, such as cell growth, differentiation, metabolism and migration.^1^

Traditionally, newly synthesized proteins can be visualized by techniques including autoradiography (AR) with metabolic incorporation of radioisotope labelled amino acids^2^ and Raman scattering (RS) microscopy with metabolic incorporation of stable isotope labelled amino acids.^3^ Recently, great progress has been achieved by adopting a powerful technique named bioorthogonal noncanonical amino acid tagging (BONCAT) coupled with click chemistry to identify and visualize newly synthesized proteins.^4^ However, these methods lack selectivity, and can't visualize a specific protein of interest (POI), as newly synthesized, *in situ*. Recently, the Schuman group expanded this technology to identify newly synthesized POIs by combining BONCAT with the proximity ligation assay (PLA).^5^

Other methods for the visualization of newly synthesized proteins include the use of genetically encoded tools. For example, Suzuki and colleagues tagged a target protein with green fluorescent protein (GFP) to construct a transmembrane Förster resonance energy transfer (FRET) pair for the detection of glycoforms of a specific glycoprotein.^6^ In addition, Chen's group exploited a protein encoded method based on enzyme-catalyzed probe ligation, to image the specific protein of glycans on live cells.^7^ Unfortunately, their method failed to image newly synthesized endogenous proteins or proteins which were not amenable to use with genetically encoded tools. Only limited progress has been achieved in this area.

As antibodies can be combined with a specific epitope in a POI with great specificity, they are particularly suitable for labelling specific endogenous proteins. Schuman's group adopted a PLA-based strategy that could identify two antibodies adjacent to each other: one detected a newly synthesized protein and the other identified a specific epitope in a POI.^5^ Here we refine the intramolecular distance resolution achieved by Tom Dieck *et al.* by using FRET. We note that the PLA technique can ligate two antibodies at distances within 40 nm,^8^ which may increase the probability of ligating two intermolecular antibodies (tags with different proteins), and thus false positives could not be avoided.

Herein, we report a FRET-based strategy for imaging specific newly synthesized endogenous proteins. The FRET acceptor is combined with the newly synthesized protein *via* click chemistry, and the FRET donor with the POI *via* an antibody. Since the FRET signal can only be detected when the distance between donor and acceptor is within 10 nm, the two fluorophores identified are more likely to be intramolecular rather than intermolecular. Although the direct observation of FRET signals was easily achieved in our study, the photobleaching based FRET efficiency imaging mode and the fluorescence lifetime imaging mode offered higher sensitivity than direct observation of FRET signals, enabling it to show the distribution of newly synthesized endogenous POI in a more convincing manner.

## Results and discussion

### Design of an intramolecular FRET experiment

**Fig. 1:**
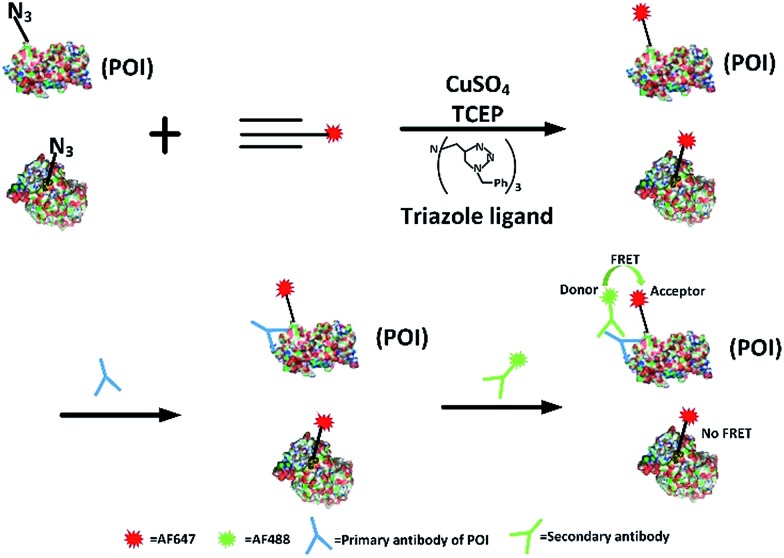
Schematic of the FRET-based methodology for visualizing newly synthesized specific proteins *in situ*. The working principle is: first, AHA is incorporated into newly synthesized proteins, then the azide-incorporated proteins are reacted with AF647-alkyne by click chemistry as FRET acceptors. A protein of interest then undergoes recognition by a specific protein antibody (blue), and is then coupled to AF488 secondary antibodies (green) as a FRET donor. Since the occurrence of FRET requires two fluorophores to be within a maximum distance of 10 nm, only an acceptor and donor on the same protein are excited through intramolecular FRET, while excess acceptors bound to other proteins are not involved.

Firstly, we attempted to design an intramolecular FRET strategy that could image newly synthesized specific proteins (Fig. 1): azidohomoalanine (AHA), serving as a surrogate for methionine, was used to tag newly synthesized proteins. To install the FRET acceptor, the Alexa Fluor (AF) 647-based tag was added by click chemistry to the newly synthesized proteins. Then a primary antibody was tagged to the specific epitope in a protein of interest (POI) in order to recognize a specific POI. Finally, a FRET donor was introduced through AF488 secondary antibodies coupled to a primary antibody. When the distance between the donor and the acceptor was within 10 nm, the specific newly synthesized endogenous proteins could be visualized by FRET signals.

FRET signals were mainly determined by the distance between the donor and the acceptor, as well as the overlap between the donor emission and acceptor absorption spectra. As we know, the POI contains a number of acceptors (methionine) which makes the distance between the donors and the acceptors suitable for effective FRET (1–10 nm). Since the donor could also be labelled on an original POI and the acceptor could be tagged on all newly synthesized proteins, the excitation spectra of a FRET pair need to be reasonably separated so as to minimize the non-FRET emission of the acceptor. It has been found that AF488 and AF647 met that demand^7^ so the problem of bleed-through in the FRET channel is avoided. Therefore, AF488 and AF647 were chosen in this work. Moreover, in our strategy, the employed small-molecule fluorophores are superior to fluorescent proteins in terms of brightness and photostability and smaller in volume.

### Imaging newly synthesized TDP-43 by FRET

**Fig. 2:**
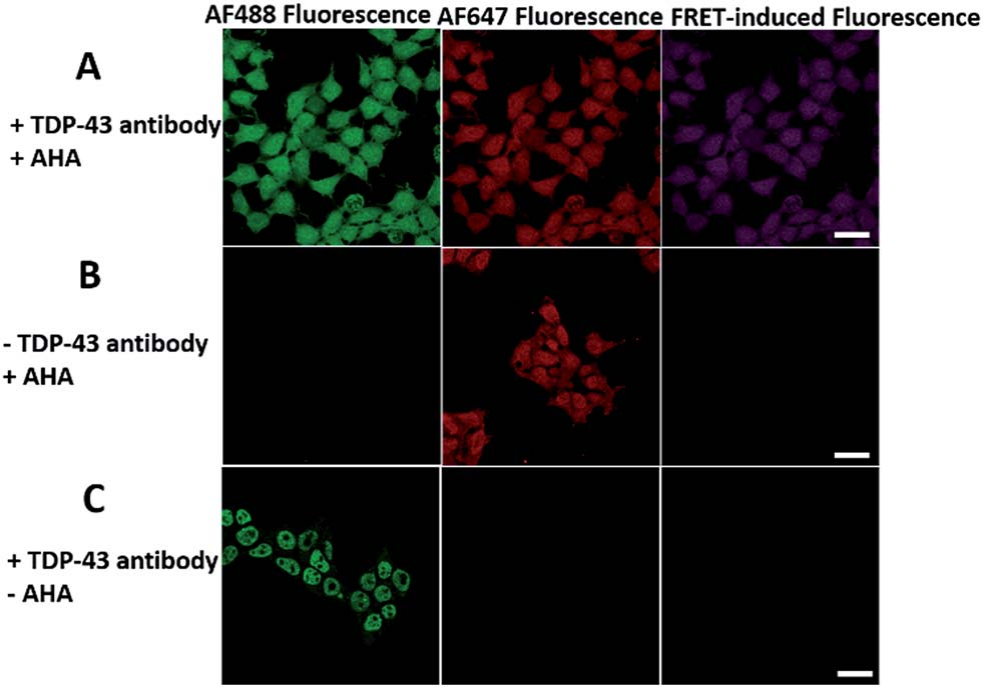
The imaging of newly synthesized TDP-43 proteins using the FRET-based method. (A) HEK293T cells were incubated with 2 mM AHA for 4 h and dually labelled with both donor and acceptor. Scale bar: 20 μm. (B) A negative control in which the antibody is absent. Scale bar: 20 μm. (C) A negative control in which AHA is absent. Scale bar: 15 μm.

We first investigated whether this FRET strategy can be used to visualize newly synthesized endogenous TAR DNA-binding protein 43 (TDP-43). TDP-43 was considered as the main component of the proteinaceous inclusions, and is closely related to amyotrophic lateral sclerosis (ALS) and frontotemporal lobar degeneration (FTLD).^9^ The outstanding characteristic of TDP-43 is nuclear localization. Nevertheless, TDP-43 proteinopathies could result in the mislocalization of the cognate protein to the cytoplasm, leading to loss of its normal nuclear localization.^10^ Thus, it is highly desirable to visualize newly synthesized endogenous TDP-43 which is associated with a wide range of neurodegenerative diseases, including Alzheimer's disease. HEK293T cells were labelled with AHA for 4 h, then the donor and acceptor were installed in the newly synthesized endogenous TDP-43. The FRET signal for the newly synthesized TDP-43 was observed only in dual-labelled donor and acceptors (Fig. 2A). None of the negative controls without the TDP-43 antibody or AHA showed FRET signals (Fig. 2B and C).

**Fig. 3:**
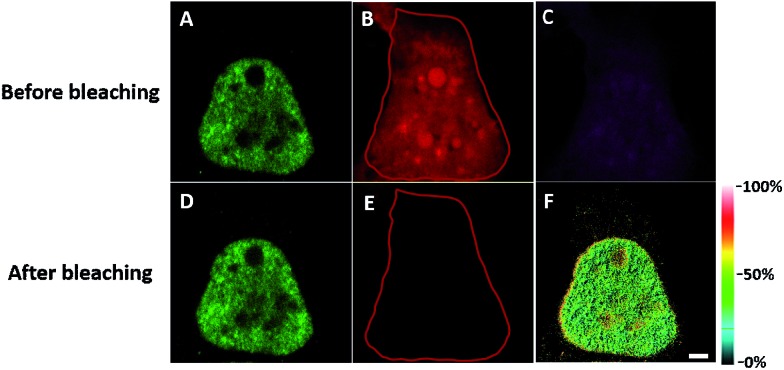
The imaging of FRET efficiency is obtained by a photobleaching experiment compared to the direct observation of FRET signals. HEK293T cells were incubated with 2 mM AHA for 4 h. (A) An image of AF488 fluorescence before photobleaching. (B) An image of AF647 fluorescence before photobleaching. (C) An image of FRET-induced fluorescence before photobleaching. [FRET-induced fluorescence (excitation: 488 nm/emission 640 to 700 nm)] (D) An image of AF488 fluorescence after photobleaching. (E) An image of AF647 fluorescence after photobleaching. The region circled by a red line indicates the area photobleached. (F) FRET efficiency imaging of newly synthesized TDP-43 proteins after photobleaching. The FRET efficiency for each pixel is measured by donor dequenching after acceptor photobleaching. Scale bar: 3 μm.

In order to see the newly synthesized TDP-43 and all proteins more clearly, we chose one cell to observe. The TDP-43 mainly existed in the nucleus because it is a nuclear localization protein (Fig. 3A). All the newly synthesized proteins existed both in the nucleus and the cytoplasm (Fig. 3B). As the overlap between the emission spectrum of AF488 and excitation spectrum of AF647 was small, the FRET signal was relatively weak, leading to difficulty in visualizing the newly synthesized TDP-43. Thus, we performed an acceptor photobleaching experiment to further confirm that the intramolecular FRET signals came from the newly synthesized TDP-43 in a more convincing manner (Fig. 3). The cell was exposed to a maximal laser light at 633 nm to bleach the fluorescence of AF647. After bleaching, the emission from AF488 was found to be increased (Fig. 3D) as a consequence of preventing FRET. The FRET efficiency estimated from the acceptor photobleaching results was 34% (Fig. 3F). As shown in Fig. 3F, the FRET efficiency image could show the distribution of newly synthesized TDP-43 more accurately compared to the direct observation of FRET signals (Fig. 3C). Moreover, a small portion of the newly synthesized TDP-43 in the cytoplasm was clearly observed. These results demonstrated that the FRET efficiency imaging mode could show newly synthesized TDP-43 mainly localized in the nucleus, which was more accurate than the direct observation of FRET signals.

**Fig. 4:**
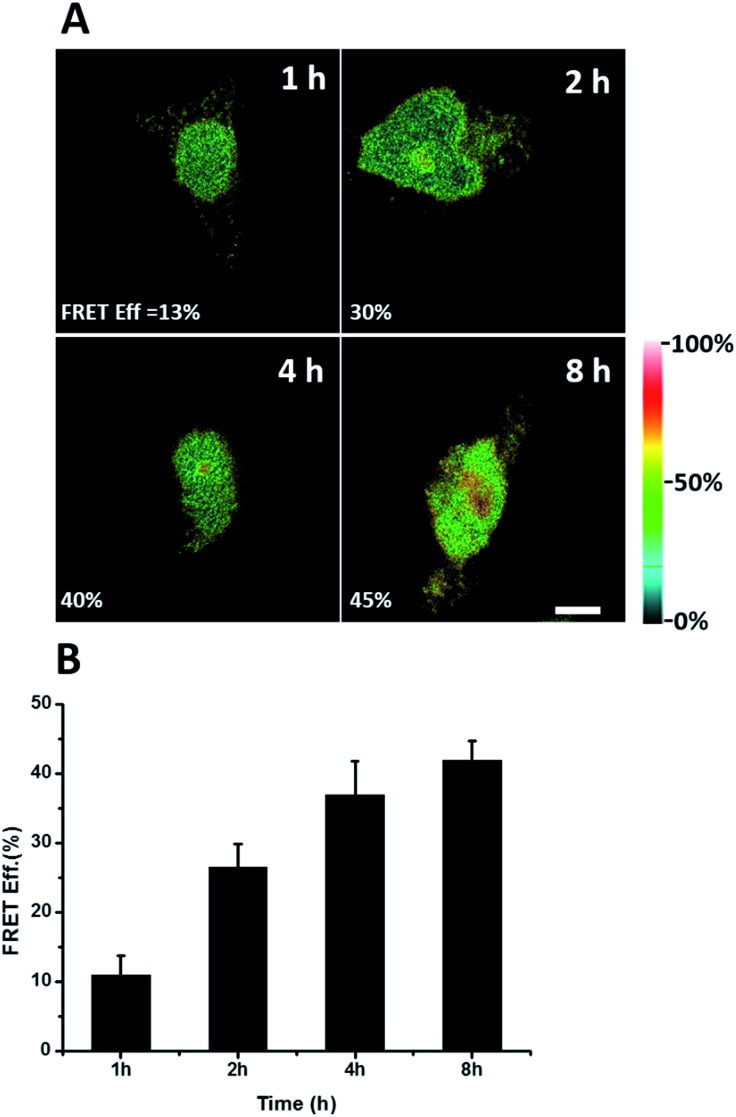
FRET efficiency imaging of newly synthesized TDP-43 proteins. (A) The FRET efficiency imaging dependence on incubation time with AHA. Scale bar: 5 μm. (B) Statistical data of the FRET efficiency measured at different time periods. An average of the FRET efficiency measured on 5 individual cells from three replicate experiments. Data are normalized, mean ± s.e.m.

We further explored newly synthesized endogenous TDP-43 *in situ* at different intervals by carrying out acceptor photobleaching experiments. Cells were labelled with AHA for 1, 2, 4 or 8 h, respectively (Fig. 4A and S1†). At the beginning, as indicated in Fig. 4A, newly synthesized endogenous TDP-43 was mainly localized in the cytoplasm, indicating that this protein may be just synthesized in the ribosome. As time passed, more TDP-43 proteins were synthesized. The FRET efficiency measured at different periods increased as time evolved (Fig. 4B). This result demonstrates that more TDP-43 was synthesized as time passed by and newly synthesized TDP-43 could be transported to the nucleus, which was consistent with the unique nuclear localization features of normal TDP-43.

### Imaging newly synthesized tubulin and CaMKIIα by FRET

**Fig. 5:**
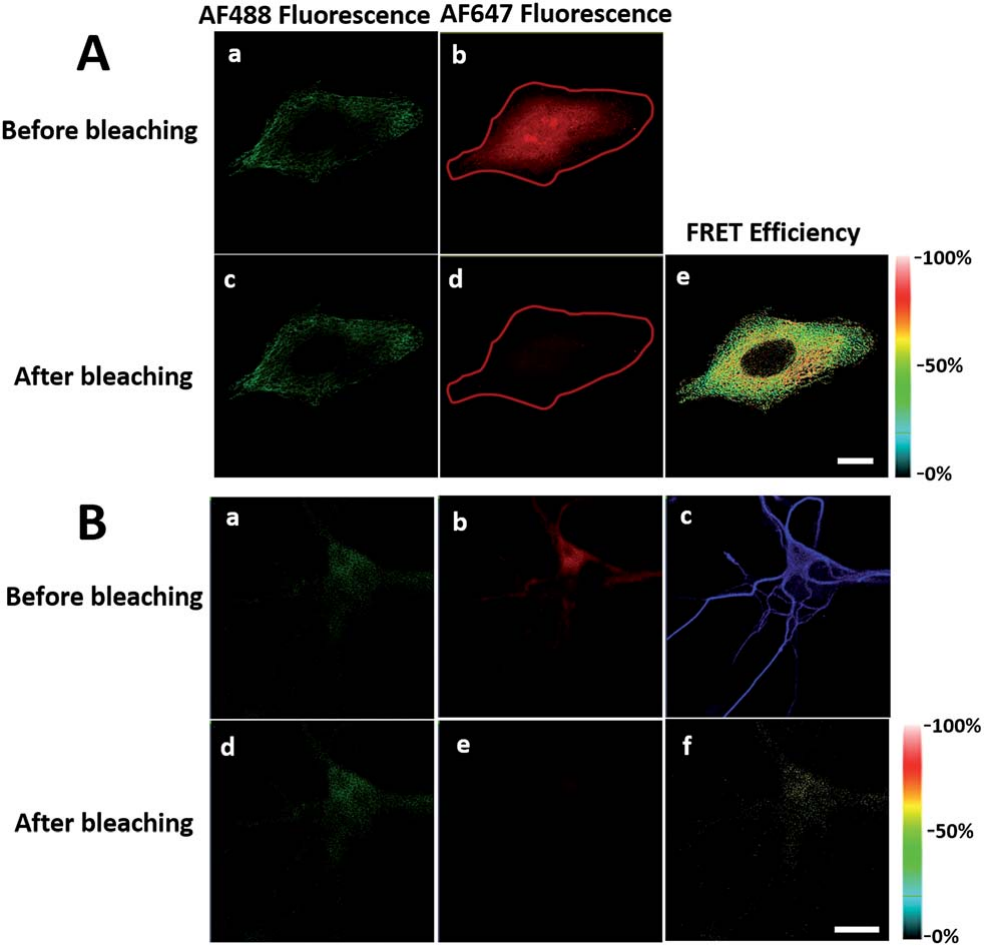
Imaging newly synthesized tubulins and CaMKIIα by FRET. (A) Imaging of FRET efficiency is obtained by a photobleaching experiment. HeLa cells were labelled with AHA for 4 h. (a) and (b) Images before bleaching. (c) and (d) Images after bleaching. The region selected by a red closed line indicates the area photobleached. (e) FRET efficiency imaging of newly synthesized tubulin. Scale bar: 10 μm. (B) FRET efficiency imaging of newly synthesized CaMKIIα in rat hippocampal neurons. Rat hippocampal neurons were labelled with AHA for 2 h. (a) and (d) Images of total CaMKIIα by using anti-CaMKIIα before and after photobleaching, respectively. (b) and (e) Images of total newly synthesized proteins before and after photobleaching, respectively. (c) Image of MAP2 to outline the profile of the soma and dendrites (AF405 fluorescence). The whole region imaged is photobleached. (f) FRET efficiency imaging of newly synthesized CaMKIIα. Scale bar: 20 μm.

To further demonstrate the utility of this method, we extend the FRET strategy to visualize other newly synthesized proteins, such as tubulins, which are major structural proteins in eukaryotic cells, essential in cell division and are important targets for cancer drugs.^11^ Senger and co-workers separated newly synthesized tubulins that were labelled with [^35^S] methionine from other proteins by immunoprecipitation and quantified by phosphor imaging.^12^ However, they failed to image the newly synthesized tubulins *in situ* at subcellular resolution. Here, we used a FRET strategy to overcome this problem. HeLa cells were treated with AHA (4 h) to pulse-label a population of newly synthesized proteins. Newly synthesized tubulins would then install the acceptor by click chemistry and the donor by immunocytochemistry. Firstly, we collected the fluorescence emission spectrum when excited at 488 nm in the cells (Fig. S2†). An emission peak at 667 nm was clearly observed in cells with dual-labelled donor and acceptor (Fig. S2, spectrum (a)†). No FRET signals were observed in the absence of donor (Fig. S2, spectrum (c)†) or acceptor (Fig. S2, spectrum (b)†). Comparing the emission peak of Alexa Fluor 488, the sample in the absence of an acceptor (Fig. S2, spectrum (b)†) was higher than that of the dual-labelled donor and acceptor (Fig. S2, spectrum (a)†). These results further demonstrated the successful energy transfer from donor to acceptor, and the fact that the acceptor spectral bleed-through in the FRET channel was avoided by choosing a FRET pair with well-separated excitation spectra. Next, we used the photobleaching method to image newly synthesized tubulins *in situ* (Fig. 5A). After bleaching, the emission from AF488 increased (Fig. 5A-c) and the FRET efficiency was estimated as 37% (Fig. 5A-e). A small selected region of the cell under the red laser (633 nm) was also investigated. The time series images of Alexa Fluor 488 showed an increase in donor emission in the photobleaching region (Fig. S3†). Taking all these findings together, it is easy to draw the conclusion that the FRET strategy could be used to visualize newly synthesized tubulins and FRET efficiency imaging could accurately show the distribution of newly synthesized tubulins that are mainly located in the microtubules in the cytoplasm.

We also used the proposed method to visualize another newly synthesized endogenous protein, namely an α subunit of Ca^2+^–calmodulin-dependent protein kinase II (CaMKIIα), in rat hippocampal neurons. CaMKIIα is the most abundant protein in the postsynaptic density, which likely contributes to the enhancement of synaptic strength. CaMKIIα is regulated by neuronal activity with respect to its subcellular localization.^13^ In agreement with the abundance of CaMKIIα mRNA in dendrites, the FRET signal for the newly synthesized CaMKIIα was found spread over both soma and dendrites, (Fig. 5B). U-118MG cells without detectable CaMKIIα were also used to prove the specificity of this method (Fig. S4†). Further protein synthesis inhibition experiments (Fig. S5†) confirmed that the FRET signals were from the newly synthesized specific proteins.

### Imaging newly synthesized proteins by FLIM

**Fig. 6:**
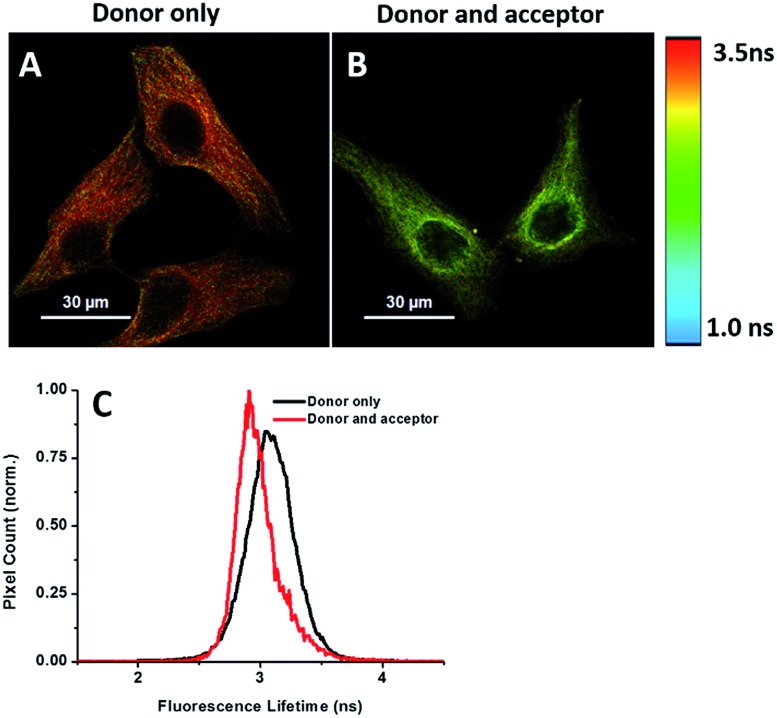
FLIM imaging of newly synthesized tubulin in HeLa cells. (A) Cells were treated with AHA for 2 h and then labelled with the donor only. (B) Cells were treated with AHA and then labelled with both donor and acceptor. (C) Histogram of *τ* values for cells treated with the donor only (black) or both donor and acceptor (red).

Energy transfer between the donor and acceptor fluorophores results in a decrease of donor fluorescence lifetime,^14^ indicative of newly synthesized proteins. We sought to visualize newly synthesized proteins that utilize the decrease in fluorescence lifetime of the donor fluorophore associated with FRET by fluorescence lifetime imaging microscopy (FLIM). This time-dependent property is advantageous since FRET efficiency is not affected by a large difference between the number of donors and acceptors and their spectral crosstalk. Cells were treated with AHA. We observed that cells treated only with the donor (tubulin primary antibody and fluorescent secondary antibody) showed a characteristic *τ* (fluorescence lifetime) value of 3.16 ns (Fig. 6A and C). When treated with both donor and acceptor, the average *τ* value decreased to 2.85 ns (Fig. 6B and C), which was consistent with FRET between AF488 and AF647 that mainly exist in microtubules within the cytoplasm. We also used this method to test newly synthesized TDP-43. The nuclei *τ* values of double-labelled donor and acceptor decreased obviously compared with that of only the donor (Fig. S6A and B†), as evident by green clusters, which may indicate that the amount of newly synthesized TDP-43 in the nucleus was higher than that in other subcellular regions. Analyzing the fluorescence lifetime imaging images, we observed that the newly synthesized tubulin concentrated in the cytoplasm and TDP-43 concentrated in the nucleus, which was consistent with the above photobleaching results.

### Verification of the intramolecular FRET-induced fluorescence

**Fig. 7:**
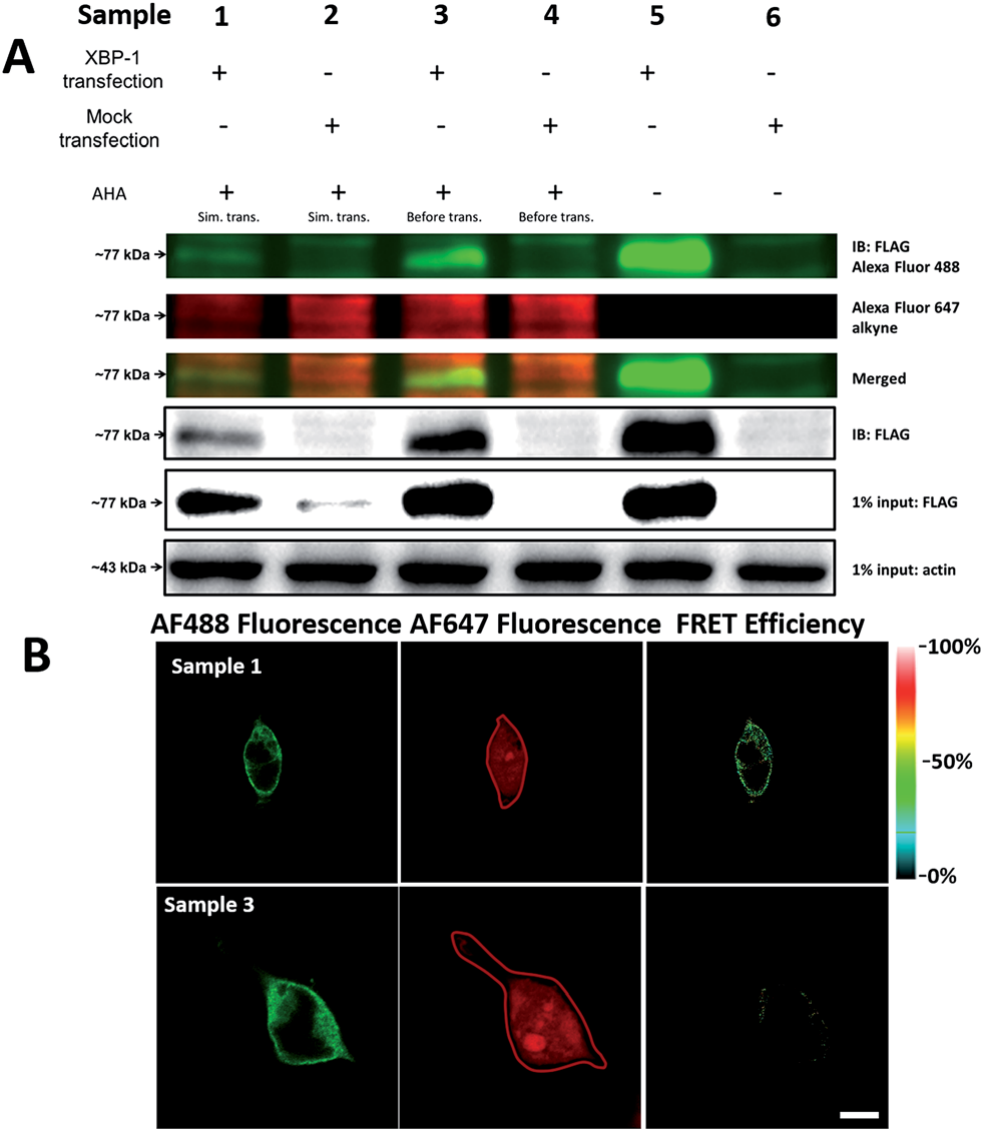
Verification of AHA integration into newly synthesized FLAG-XBP1. (A) HEK293T cells were transfected with FLAG-XBP1 expressing plasmid (samples 1, 3 and 5) or control plasmid (samples 2, 4 and 6), simultaneously with culturing in AHA-containing media (samples 1 and 2), after culturing in AHA-containing media (samples 3 and 4), or without culturing in AHA-containing media (samples 5 and 6). Cell lysates were treated with click reaction reagents and immunoprecipitation. (B) The imaging of newly synthesized XBP1 proteins using the photobleaching method. Sample 1: the HEK293T cells were transfected with expression plasmids for XBP1 with medium containing AHA for 10 h. The cells were labelled with both donor and acceptor. Sample 3: cells were firstly cultured in medium with AHA for 10 h. Then cells were changed to fresh medium not containing AHA and transfected with expression plasmids for XBP1 for 10 h. The region enclosed by a red line indicates the area photobleached. Scale bar: 10 μm.

It was demonstrated above that FRET signals could be detected by the above methods. Nevertheless, whether the signals came from intramolecular FRET on the same protein needs to be further explored. We expressed an exogenous protein with a FLAG (DYKDDDDK)-tag that could specifically bind to a donor, then labelled the specific exogenous protein on the donor, or both the donor and acceptor, and lastly the photobleaching method was used to demonstrate that the FRET-induced fluorescence was intramolecular. The mammalian unfolded protein response protected the cell against the stress of misfolded proteins in the endoplasmic reticulum (ER). X-Box binding protein-1 (XBP1) regulates a subset of ER resident chaperone genes in the unfolded protein response.^15^ HEK293T cells were transfected with FLAG-XBP1 expressing plasmid (samples 1, 3 and 5) or control plasmid (pSQT1313) (samples 2, 4 and 6), simultaneously with AHA media culturing (samples 1 and 2), after AHA media culturing (samples 3 and 4) or without AHA media culturing (samples 5 and 6), respectively. In theory, newly synthesized exogenous XBP-1 with FLAG could not be labelled with AHA in the control experiment. In sample 1, as shown by the immunoprecipitation result in Fig. 7A, the intensity of AF488 is higher than those in samples 2, 4 and 6, and the intensity of AF647 is higher than those in samples 5 and 6, which indicated successful FLAG-XBP1 expression and AHA integration. Also, highly similar spatial changing trends of AF488 and AF647 intensities along the linear ROI indicate that AHA was successfully integrated into newly synthesized FLAG-XBP1 (Fig. S7†). In sample 3, the intensity of AF488 is much higher than those in samples 2 and 4, while the intensity of AF647 is similar to those in samples 2 and 4, which shows that the enrichment of FLAG-XBP1 didn't contribute to the detection of AF647. Spatial changes of AF488 and AF647 along the linear ROI didn't show highly similar trends. These all suggest that FLAG-XBP1 was successfully expressed in sample 3 and barely AHA-integrated. The intensities of AF488 in samples 1 and 3 are lower than those in sample 5, since culturing in media supplemented with dialyzed-FBS may influence protein synthesis. As expected, the FRET efficiencies of the cells dual-labelled (Fig. 7B, sample 1) and only labelled with the donor (Fig. 7B, sample 3) were 35% and 7% respectively (Fig. S8†). The FRET efficiency signal for the newly synthesized XBP-1 was mainly in the cytoplasm, which is consistent with the abundance of XBP1 mRNA in endoplasmic reticulum.

**Fig. 8:**
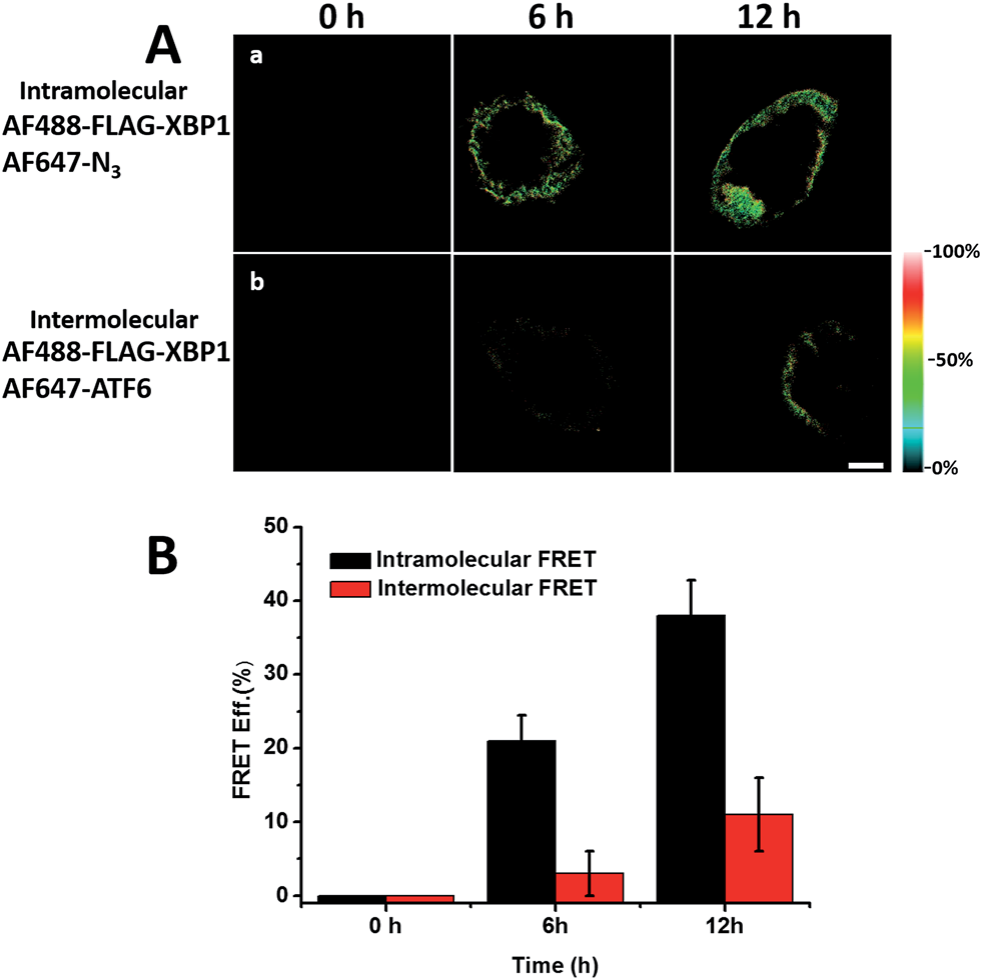
FRET efficiency imaging of intramolecular newly synthesized proteins or intermolecular newly synthesized proteins. (A) (a) HEK293T cells were cultured in AHA media and transfected with FLAG-XBP1 expressing plasmid simultaneously, and were fixed after 6 and 12 hours. Untreated cells were used as a 0 hour negative control. Cells from different time points were stained with anti-FLAG primary antibody and donkey anti-mouse Alexa Fluor 488 secondary antibody. Alexa Fluor 647 alkyne was labelled to detect all newly synthesized proteins. (b) Anti-ATF6 primary antibody and goat anti-rabbit Alexa Fluor 647 secondary antibody were used to detect the ATF6 protein, which is the strongest interaction partner indicated by the STRING database. (B) Statistical results of the FRET efficiency. Averages of the FRET efficiencies measured on 5 individual cells from three replicate experiments. Data are normalized means ± s.e.m.

We sought to further demonstrate that FRET signals detected from labelled proteins are mainly intramolecular, based on immunostaining of XBP1 and its interaction partner activating transcription factor 6 (ATF6). As shown in Fig. 8, FRET analysis results showed that intra-molecular FRET efficiency increased rapidly during the 12 hour treatment, while inter-molecular FRET efficiency remained at a low level, although somewhat increased. This indicated that the FRET signal detected in AF647 alkyne-labelled cells was mainly intra-molecular.

## Conclusions

In this study, we present a FRET-based method for newly synthesized specific protein imaging by installing a donor through antibodies and an acceptor through click chemistry. We found that the photobleaching based FRET efficiency imaging mode and the fluorescence lifetime imaging mode showed the distribution of newly synthesized proteins with higher sensitivity compared to direct FRET signals imaging. We demonstrated our strategy by imaging newly synthesized TDP-43 in HEK293T cells, tubulins in HeLa cells and CaMKIIα in rat hippocampal neurons. Furthermore, our imaging revealed the turnover of newly synthesized protein endogenous TDP-43 in the HEK293T cells at different intervals. In theory, as long as the antibodies of a POI are available, we can investigate the distribution of the newly synthesized endogenous proteins. This powerful tool may provide new insight into tracking POI synthesis and redistribution in their native cellular context.

## Experimental section

### Cell culture

HeLa and HEK293T cells were cultured with Dulbecco's modified Eagle's medium supplemented with 10% FBS, 1% penicillin–streptomycin solution (100 μg mL^–1^) at 37 °C in a humidified atmosphere containing 5% CO_2_.

### Hippocampal neurons

Briefly, we dissected hippocampi from postnatal day 0–1 rat pups of either sex, dissociated them with 0.25% trypsin–EDTA and plated them onto poly(d-lysine)-coated glass-bottom Petri dishes (NEST) at a density of 40 × 10^3^ cells per cm^2^. Hippocampal neurons were nurtured in a humidified atmosphere at 37 °C and 5% CO_2_ in growth medium (Neurobasal-A supplemented with B-27 and GlutaMAX, Life Technologies).

### Metabolic labelling with AHA

Cells were incubated in methionine-free medium supplemented with dialyzed FBS for 30 min to deplete endogenous methionine. For AHA labelling, we supplemented methionine-free medium with 2 mM AHA and dialyzed FBS. After incubation for a certain period of time, the cells were washed with PBS on ice to remove excess amounts of AHA, then immediately fixed by chilled 4% paraformaldehyde for 20 min. Then, a CuAAC reaction mixture containing 200 μM triazole ligand tris((1-benzyl-1*H*-1,2,3-triazol-4-yl)methyl)amine (TBTA), 2 μM AF647-alkyne, 400 μM TCEP and 200 μM CuSO_4_ was added to the cells, followed by incubation in a humid box overnight at 20 °C with gentle agitation. Then we washed the cells with 1% Tween-20 in PBS three times. After that, cells were washed with PBS for three times before immunostaining.

### Microscopic analysis and FRET measurement

Cell fluorescence and FRET imaging were conducted on a Zeiss LSM 780 laser scanning confocal microscope equipped with a 63× oil immersion objective lens (N.A. 1.4). A 488 nm line of gaseous argon laser could excite Alexa Fluor 488 fluorescence so as to collect a spectrum with a 493 to 630 nm band-pass filter. By using a 633 nm line of gaseous argon laser, Alexa Fluor 647 fluorescence was excited to collect a spectrum with a 640 nm long-pass filter. The fluorescence emission spectra of cells were taken using the gaseous argon laser (excited at 488 nm). The emission was collected from 510 to 695 nm using a 3 nm step. Acceptor photobleaching was carried out also using the Zeiss LSM 780 laser scanning confocal microscope. Images of AF488 and AF647 were obtained first. Then the cell was placed under 633 nm laser light at 100% intensity to bleach the AF647 until the intensity dropped to 5% of the original. Between every two scans, each pixel was exposed to laser irradiation for 1.5 μs each time and 50 times repeatedly. Then, the average increase in the donor (AF488) fluorescence in the cell after AF647 photobleaching was measured. The efficiency of energy transfer (*E*) was given by the following equation: *E* = 1 – *F*_DA_/*F*_D_, where *F*_D_ and *F*_DA_ are the relative fluorescence intensities of the donor in the absence (*F*_D_) or presence (*F*_DA_) of the acceptor.

### Fluorescence lifetime imaging

Time-correlated single photon counting (TCSPC) data sets were acquired on an inverted OLYMPUS FV1200 microscope with a 40×/0.95 NA lens equipped with a Picoquant picoHarp 300 (Germany) controller. Samples were excited by picosecond 485 nm pulses generated by a 40 MHz laser. The non-descanned emission was collected from a 520/35 nm bandpass filter (PicoQuant, Germany) and detected by a MPD SPAD detector (PicoQuant, Germany). Images of 512 × 512 pixels were obtained. Photon data were analyzed using SymPhoTime 64 image software. Analysis of the acquired fluorescence lifetime imaging microscopy (FLIM) data was performed by first binning (3 pixels × 3 pixels) the time dependent photon image and assigning a minimum threshold count of 50 recorded photons for modelling. Fluorescence lifetime values derived from the exponential fits are then displayed as a heat-map image.

### Plasmid transfection and AHA labeling

Samples were prepared as follows: media of HEK293T cells in samples 1 and 2 were replaced with DMEM containing 10% dialyzed FBS and 2 mM AHA, and the cells were transfected with FLAG-XBP1 expressing plasmid and control plasmid (pSQT1313) using Lipofectamine 3000 (Thermo, L3000015), respectively, then cultured for 10 hours. Cells of samples 3 and 4 were cultured in DMEM containing 10% dialyzed FBS and 2 mM AHA for 10 h, then the media were replaced with Met-free DMEM for 30 min, followed by normal DMEM, and the cells were transfected with XBP-1 expressing plasmid and control plasmid, respectively, incubated for 10 h. Cells of samples 5 and 6 were transfected with XBP-1 expressing plasmid and control plasmid, respectively, followed by 10 h incubation, without culturing in AHA-containing DMEM.

### Click reaction and immunoprecipitation

The cells were lysed with 0.5 mL ice-cold 1% (w/v) SDS in PBS by vigorous vortexing, boiled for 10 min at 96 °C and chilled on ice. Then 4.5 mL ice-cold PBS, 50 μL 20% (v/v) Triton-X100, 5 μL 2 M MgCl_2_ and 0.2% (v/v) BitNuclease were added to each sample, followed by continuous mixing at 4 °C for 1 h. Cell lysates were collected after centrifugation at 2000*g* for 7 min at 4 °C to remove cell debris, then incubated with the click reaction mixture overnight at 4 °C. FLAG-XBP1 protein was immunoprecipitated with anti-FLAG affinity gel. The agarose beads were washed four times with PBS-Tween (PBST), followed by heating in SDS-loading buffer to release the proteins.

### Fluorescent and chemiluminescent immunoblotting

For fluorescent immunoblotting, proteins separated by SDS-PAGE were transferred to low-fluorescence PVDF membrane (Abcam, ab133411), blotted by anti-FLAG rabbit polyclonal primary antibody (MBL, PM020, 1 : 2000) followed by goat anti-rabbit Alexa Fluor 488 conjugated secondary antibody (Thermo, A11034, 1 : 200). The membrane was scanned with a ProteinSimple FluorChem M system (excitation 475 nm/537 nm filter, 26 nm band-pass for Alexa Fluor 488 and excitation 632 nm/710 nm filter, 40 nm band-pass for Alexa Fluor 647). For chemiluminescent immunoblotting, proteins were blotted by anti-FLAG rabbit polyclonal primary antibody (1 : 2000), followed by anti-rabbit-HRP conjugated secondary antibody (1 : 2000).
