# Supplementary material for: Imaging specific newly synthesized proteins within cells by fluorescence resonance energy transfer
Source: Chem Sci. 2016 Sep 12;8(1):748–54. doi: 10.1039/c6sc02610a (PMC5299820; doi:10.1039/c6sc02610a)
Supplement: Supplementary file 1 [file SC-008-C6SC02610A-s001.pdf]

Electronic Supplementary Material (ESI) for Chemical Science.
This journal is © The Royal Society of Chemistry 2016

Supporting Information

Imaging specific newly synthesized proteins within cells by fluorescence resonance energy transfer

Linfeng Sheng, Lesi Cai, Jie Liu, Sichun Zhang, Jing-Juan Xu, Xinrong Zhang, Hong-Yuan Chen

This file includes:

Experimental Section

Figure S1 to S8

Experimental section

Reagents and Antibodies. Click-iT® AHA (L-azidohomoalanine), Neurobasal®-A Medium and Alexa Fluor® 647 Alkyne (AF647-alkyne) were purchased from Invitrogen (USA). Tris (2-carboxyethyl) phosphine hydrochloride (TCEP), Tris((1-benzyl-1H-1,2,3-triazol-4-yl)methyl)amine (TBTA), Tween-20, Triton X-100, $CuSO_4$, Albumin bovine serum (BSA) and Anisomycin from *Streptomyces griseolus* were purchased from Sigma-Aldrich Inc. (USA). Dulbecco's modified Eagle's medium (DMEM), Trypsin-EDTA, Fetal bovine serum (FBS), penicillin-streptomycin solution and phosphate buffer saline (PBS, pH 7.4) were purchased from Corning (NY, USA). The DMEM media without methionine was produced by Gibco (Cat. 21013024). The dialyzed FBS was purchased from Biological Industries. Neurobasal-A medium without methionine was purchased from BrainBits LLC. Antibodies were used in the following dilutions: Ms-anti-α-tubulin monoclonal (Sigma, T5168, 1:500), rb-anti-TDP-43 (proteintech, 10782-2-AP, 1:300), rb-anti-Camk2a (Santa Cruz Biotechnology, sc-9035, 1:1000), ch-anti-MAP2 (Abcam, ab5392, 1:2000). ATF6 Polyclonal Antibody (proteintech, 1:100). Secondary antibodies of different host for immunocytochemistry coupled to Alexa Fluor 488 were purchased from Abcam plc Co., Ltd. (USA). XBP-1 1: pFLAG.XBP1u.CMV2 was a gift from David Ron (Addgene plasmid # 21832).[1] pSQT1313 was a gift from Keith Joung (Addgene plasmid # 53370).[2]

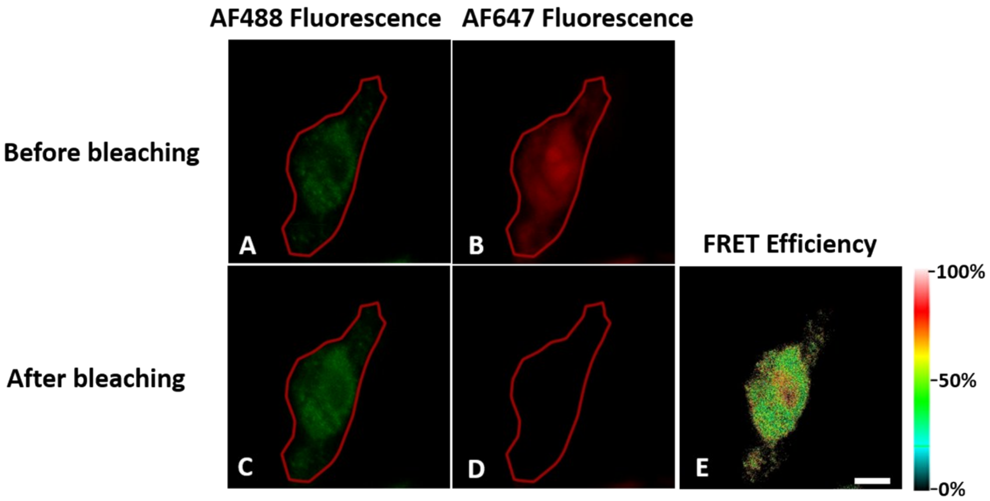

Figure S1. The imaging of FRET efficiency is obtained by photobleaching experiment. HEK293T cells were labelled with AHA for 8 h. (A) the image of AF488 fluorescence before photobleaching. (B) the image of AF647 fluorescence before photobleaching. (C) the image of AF488 fluorescence after photobleaching. (D) the image of AF647 fluorescence after photobleaching. The region enclosed by red line indicates the area photobleached. (E) FRET efficiency imaging of newly synthesized TDP-43 proteins. Scale bar: 10 μm. As idicated in Fig. S1E, the FRET efficiency image could show the distribution of newly synthesized TDP-43 accurately.

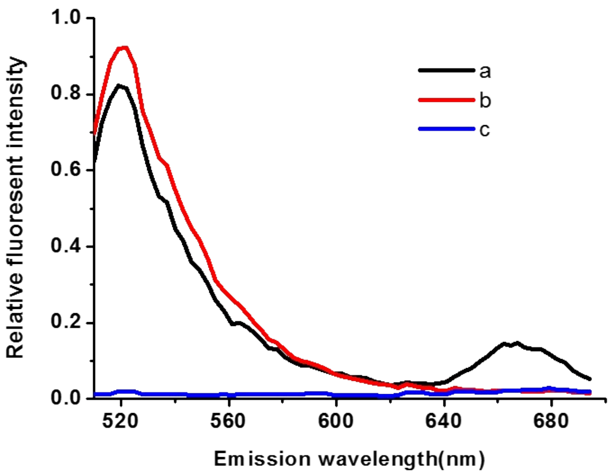

Figure S2. Fluorescence emission spectrum from cells when excited at 488 nm. (a) HeLa cells were incubated with 2 mM AHA for 4 h and dually labeled with donor and acceptor. (b) Cells were incubated without AHA and labeled with AF488 secondary antibody as donor. (c) Cells were incubated with 2 mM AHA and labeled with Alexa Fluor647-alkyne as acceptor and no donor.

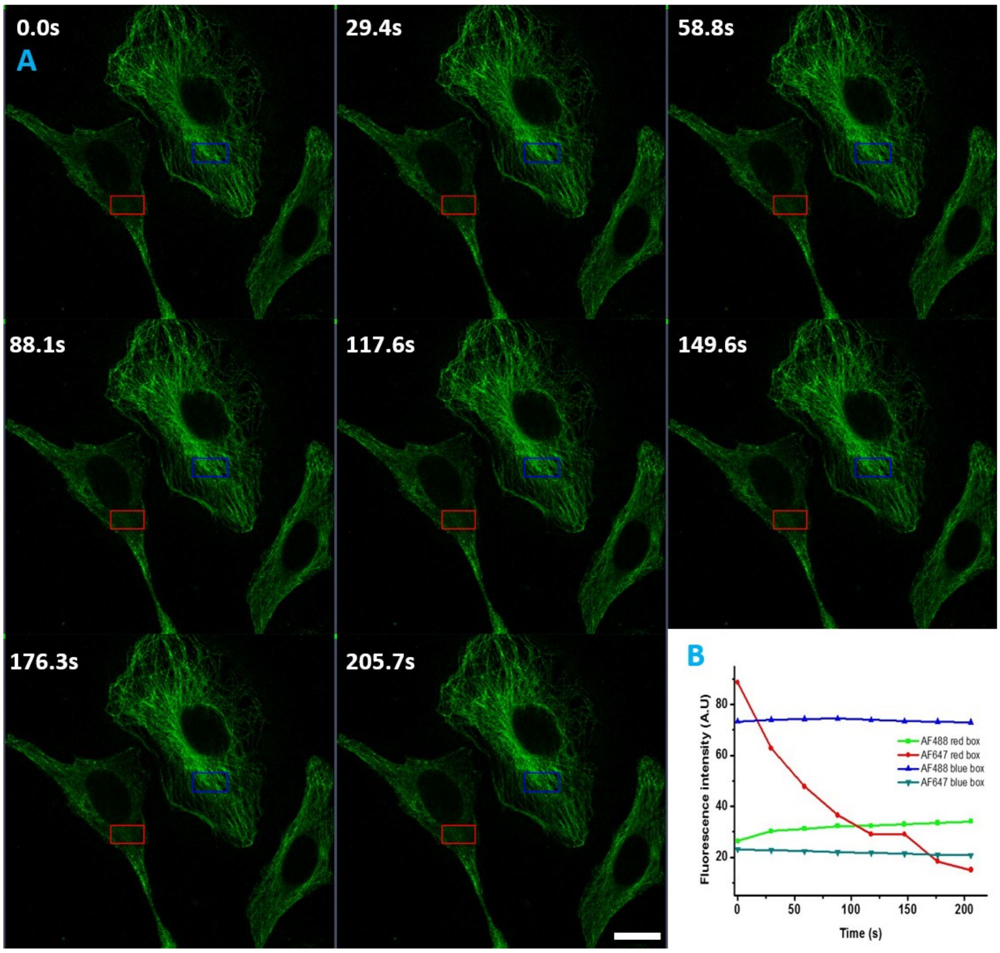

Figure S3. (A) The time series images of Alexa Fluor 488 during photobleaching. Selected region of the cell photobleached under the red laser (red box) and region not photobleached (blue box) were investigated. (B) The time series fluorescence intensity of selected region. Scale bar: 15 μm. During bleaching, the emission from AF488 was increased as a consequence of preventing FRET. The emission from AF647 was decreased as photobleaching of the red laser.

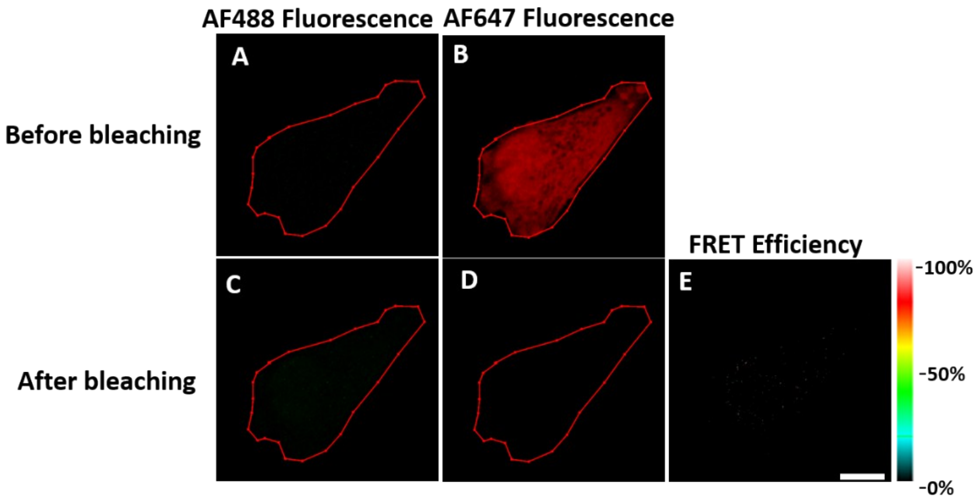

Figure S4: The imaging of FRET efficiency obtained by photobleaching experiment. U-118MG cells were labelled with AHA for 6 h. (A) the image of AF488 fluorescence before photobleaching. (B) the image of AF647 fluorescence before photobleaching. (C) the image of AF488 fluorescence after photobleaching. (D) the image of AF647 fluorescence after photobleaching. The region enclosed by red closed line indicates the area photobleached. (E) FRET efficiency imaging. Scale bar: 10 μm. Image A indicates that U-118MG cells don't have detectable CaMK II α. Image E shows that no FRET efficiency signals could be detected.

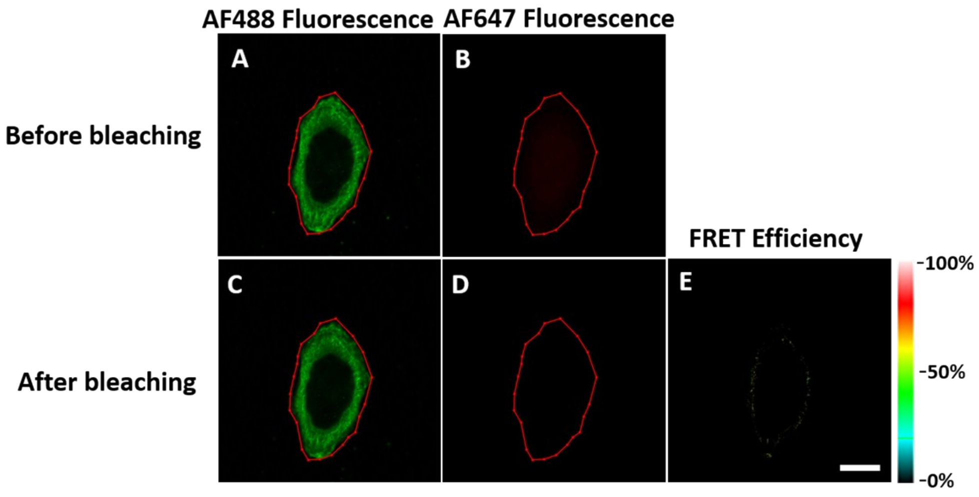

Figure S5. The imaging of FRET efficiency obtained by photobleaching experiment. HeLa cells were labelled with AHA together with 5 μM anisomycin for 10 h. (A) the image of AF488 fluorescence before photobleaching. (B) the image of AF647 fluorescence before photobleaching. (C) the image of AF488 fluorescence after photobleaching. (D) the image of AF647 fluorescence

after photobleaching. The region enclosed by red line indicates the area photobleached. (E) FRET efficiency imaging of newly synthesized tubulin. Scale bar: 10 μm. As idicated in Fig. S5E, the FRET efficiency image of newly synthesized tubulin displays near vanishing signal.

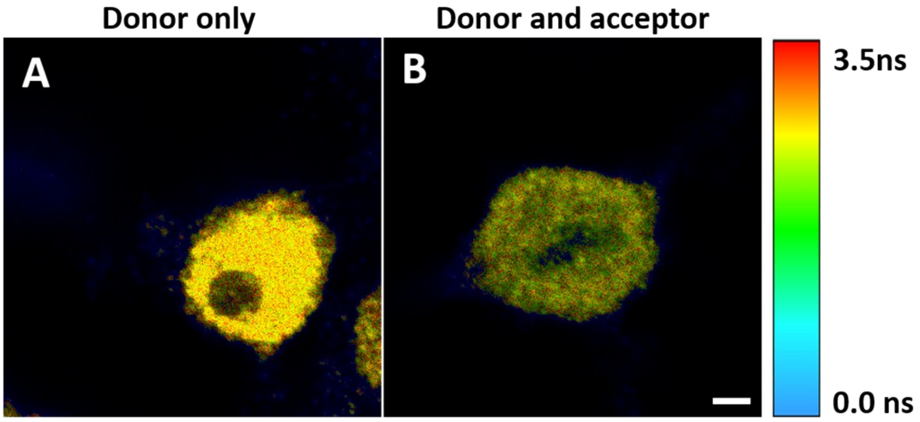

Figure S6. Fluorescence lifetime imaging of newly synthesized TDP-43 in HEK293T cells. (A) Cells treated with AHA for 2 h and then labelled with donor only. (B) Cells treated with AHA and then labelled with donor and acceptor. Scale bar: 2 μm.

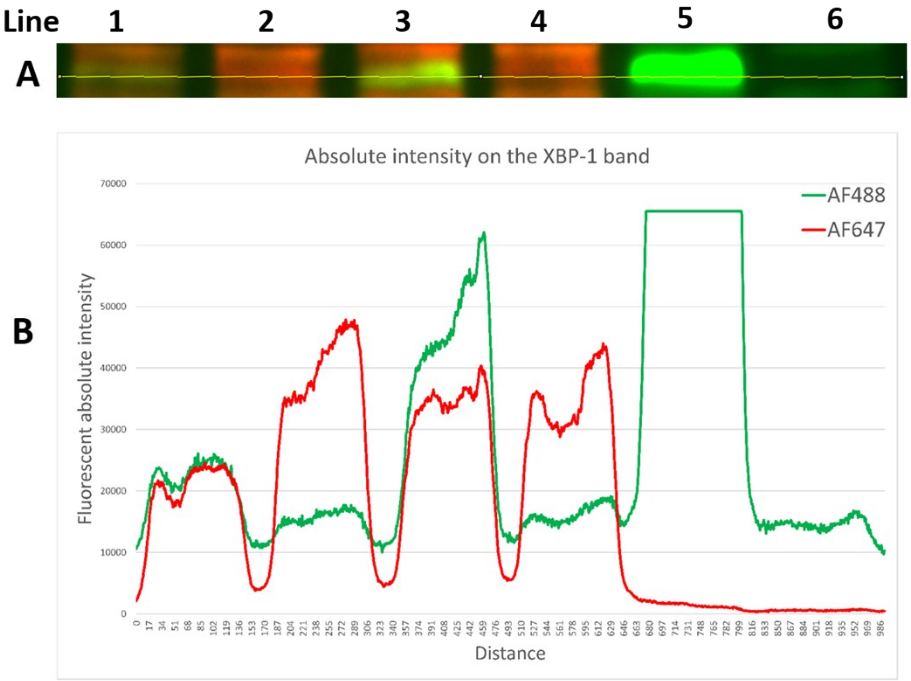

Figure S7. A line ROI was drawn through the center of the bands of FLAG-XBP1, and absolute intensities of AF488 and AF647 of the pixels on the line were measured. (A) Position of the line ROI. (B) Absolute intensity on the XBP-1 band.

In lane 1, spatial changes of AF488 and AF647 show highly similar trends, which indicated that AHA was successfully integrated into newly synthesized FLAG-XBP1. In lane 3, the intensity of AF488 is much higher than that in lane 4, while intensity of AF647 is similar to that in lane 4, which indicated that FLAG-XBP1 was successfully expressed in sample 3 without AHA integration. While intensities of lane 2, 3 and 4 were similar to each other, the intensity of lane 1 is lower than three other AHA-positive lanes. This may be due to that strong expressing of FLAG-XBP1 under CMV2 promoter and relatively poor nutrient supply of dialyzed FBS impaired newly synthesis of potential interacting AHA-labeled proteins.

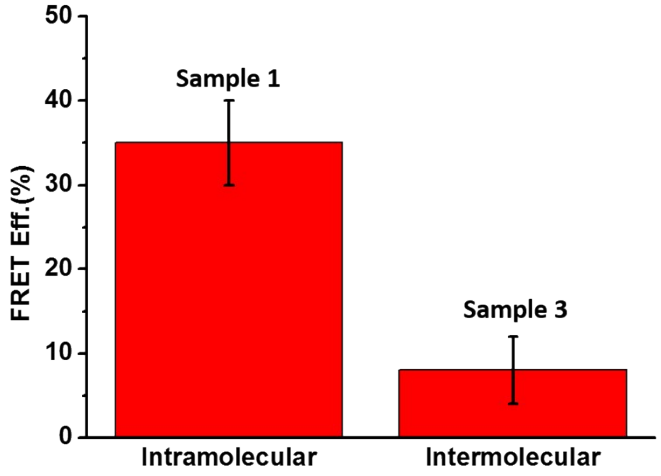

Figure S8. FRET efficiency of Sample 1 and Sample 3. Averages of the FRET efficiencies measured on 5 individual cells from three replicate experiments. Data are normalized mean ± s.e.m.

Reference:

[S1] Calfon M, Zeng H, Urano F, Till JH, Hubbard SR, Harding HP, Clark SG, Ron D. Nature. 2002, 415, 92-96

[S2] Tsai SQ, Wyvekens N, Khayter C, Foden JA, Thapar V, Reyon D, Goodwin MJ, Aryee MJ, Joung JK. Nat. Biotechnol. 2014, 32,569-576
